# Strategy of treatment in a patient with acute blunt traumatic thoracic aorta dissection

**DOI:** 10.1186/1749-8090-8-S1-P51

**Published:** 2013-09-11

**Authors:** I Yurekli, S Yazman, K Ergunes, I Peker, L Yilik, A Gurbuz

**Affiliations:** 1 Izmir Katip Celebi University Ataturk Training and Research Hospital, Department of Cardiovascular Surgery, Izmir, Turkey

## Background

Traumatic thoracic aortic dissection is potentially life-threatening, and is often fatal within the first hours. Endovascular stent-graft placement is safer, effective treatment method than conventional open surgery.

## Methods

A 37-year-old female suffered from a road traffic accident while out of the car. Her mental status was clear. She had no history of hypertension and diabetes. On admission her vital signs were stable. Systolic blood pressure was 110 mmHg. Neurological examination was normal. Upper and lower extremity pulses were palpable with hand. Right upper extremity radiography showed fracture in radial and ulnar bones. An ECG showed sinus rhythm, and did not suggest acute ischemia. Initial 2-dimensional echocardiography showed no regional wal motion abnormality and pericardial effusion. Ct angiogram (CTA) showed traumatic aortic dissection in proximal descending thoracic aorta with left pleural effusion.

## Results

Patient was transferred to the operation room. Stenting was performed using a 26x112 mm Valiant thoracic stent graft (Medtronic AVE). Postoperative aortogram showed good expansion of the stent without dye leakage. After uneventful recovery, she was discharged on day 14.

## Conclusion

Endovascular treatment for acute traumatic thoracic aortic dissection is feasible and represents a valid alternative to conventional open surgery in selected patients.

